# Posthuman Affirmative Business Ethics: Reimagining Human–Animal Relations Through Speculative Fiction

**DOI:** 10.1007/s10551-021-04801-8

**Published:** 2021-04-03

**Authors:** Janet Sayers, Lydia Martin, Emma Bell

**Affiliations:** 1 Massey Business School, Massey University, 1 University Avenue, Albany, Auckland, 0632 New Zealand; 2 University of Auckland Business School, University of Auckland, Sir Owen G Glenn Building, 12 Grafton Road, Auckland, 1010 New Zealand; 3 The Open University Business School, The Open University, Walton Hall, Milton Keynes, MK7 6AA UK

**Keywords:** Posthumanism, Speculative fiction, Imagination, Affirmative ethics, Animals, Feminism

## Abstract

Posthuman affirmative ethics relies upon a fluid, nomadic conception of the ethical subject who develops affective, material and immaterial connections to multiple others. Our purpose in this paper is to consider what posthuman affirmative business ethics would look like, and to reflect on the shift in thinking and practice this would involve. The need for a revised understanding of human–animal relations in business ethics is amplified by crises such as climate change and pandemics that are related to ecologically destructive business practices such as factory farming. In this analysis, we use feminist speculative fiction as a resource for reimagination and posthuman ethical thinking. By focusing on three ethical movements experienced by a central character named Toby in Margaret Atwood’s *MaddAddam* trilogy, we show how she is continually becoming through affective, embodied encounters with human and nonhuman others. In the discussion, we consider the vulnerability that arises from openness to affect which engenders heightened response-ability to and with, rather than for, multiple others. This expanded concept of subjectivity enables a more relational understanding of equality that is urgently needed in order to respond affirmatively to posthuman futures.

## Introduction

We live in a world dominated by crises on a global scale related to climate change, ecological degradation and destruction, and the threat of mass extinction of animals and their habitats.^1^ Businesses are suggested to be a major contributor to these crises through the ideology of profit-maximisation (Peggs, 2015). Evidence of negative human impact on the Earth’s ecology, the Anthropocene, is provided by the COVID-19 pandemic, caused by transmission of infectious diseases between species (Shah, 2017).^2^ The pandemic has also made visible and exacerbated existing human inequalities around homelessness, immigrant labour, ethnic and racial groups, women, and workers in precarious front-line service work.^3^ Consequently, health scientists claim it is “almost common sense” (Verweij & Bovenkerk, 2016, p. 1) to acknowledge the imbricated relationship between ecologies, human and nonhuman animal^4^ health, and inequality as a driver of poor human health outcomes.

Yet business ethics research has been slow to engage with the ethics of human-animal relations, even though they are well-developed in other disciplines (c.f. Giraud, 2013). Instead the field continues to be shaped by a humanist, anthropocentric perspective that positions “[m]an… as subject, at the centre” (Willmott, 1998, p. 94) and fails to question the commonplace assumption that “human beings are…the central element of the Universe” (Willmott, 2014, p. 24). Ethical considerations are reduced to seeking to balance the “business needs of profit-maximisation and shareholder value with the interests of the natural environment and local communities” (de Cock et al., 2019, p. 2). This perspective reflects an anthropocentric rationale that conceptualises animals as objects and reduces them to a consumable commodity (Crittenden, 2001; Desmond, 2010; Sayers et al., 2019).

The industrial-agricultural practice of factory farming provides a specific, biopolitical “problem space” (Labatut et al., 2016, p. 317) in which to explore human–animal relations in business ethics. As Clarke and Knights (2021, p. 3, citing Cole & Stewart, 2016) note, “we currently kill more than 150 billion nonhuman animals for the purpose of human consumption”. Factory farming is linked to climate change and ecological degradation and contributes to animal suffering on an industrial scale, potentially providing the conditions for pandemics to occur (Shah, 2017). As Narayanan (2016) states “intensive farming involves animal husbandry practices that amount to extraordinary cruelty to animals” (p. 174) as well as being a major contributing factor to the current climate emergency “well above total transport emissions, accounting for 18 per cent of the total greenhouse emissions” (p. 174). These practices are sometimes justified on the grounds that “food animals” are of lower moral status than other living beings and “eating animal flesh is a God-given prerogative” (Zuzworsky, 2001 p. 177–178). Ethical discussions of factory farming focus on animal welfare concerns (Simpson & Rollin, 1984), and the need to ensure animals do “not endure unnecessary suffering” (Maloni & Brown, 2006, p. 39). This moral imperative implies there are situations where animal suffering is ethically justified (Clarke & Knights, 2021).

In this paper we call for more imaginative responses to comprehend not only the symptoms of these current crises, but also and more importantly, the ethical philosophies and assumptions that shape interactions between human and nonhuman animals. We approach these issues by posing the question: what would a posthuman affirmative business ethics look like and what changes in thinking and practice would this involve? The paper is structured as follows: we begin by considering the role of speculative fiction in ethical imagination and introduce the concept of figurations. In the section that follows, we use the work of Rosi Braidotti (2013, 2019a) to explain how posthumanism develops a fluid, nomadic conception of the ethical subject that relies upon affective, material and immaterial connections to multiple others (Braidotti, 2011, 2013, 2019a; Wolfe, 2010). We then present an analysis of Margaret Atwood’s speculative fiction trilogy, *Oryx and Crake* (2003 [2013]), *The Year of the Flood* (2009 [2010]) and *MaddAddam* (2013 [2014]) in which we identify three ethical ‘movements’ experienced by a central character in the novels, named Toby: ‘from equality towards in-disposability’; ‘from individualism towards affect’; and ‘imagining new forms of becoming’. In the discussion, we return to the problem space of factory farming and the COVID-19 pandemic, and explain why posthuman affirmative ethics necessitates revision of ideas about equality. Through this, we aim to demonstrate the relevance and importance of posthuman affirmative ethics by inviting reconsideration of what it means to be a human subject and moving away from false oppositions between human and nonhuman animals.

## Thinking Imaginatively and Affirmatively Through Speculative Fiction

Thinking imaginatively about ethics is central to understanding and responding to ecological crises (Rozuel, 2016) which has so far been impeded by a “failure of the imagination” (Bergthaller, 2010, p. 730). The label ‘speculative fiction’ encompasses science fiction, fantasy, utopian and dystopian narratives and posthuman fiction. Atwood (2011) describes speculative fiction as stories that imagine “other worlds located somewhere apart from our everyday one” in order to “free the human imagination” and push it to its limits (pp. 8, 41, 62). De Cock (2009) contends that realist literature (both fiction and non-fiction) can paralyse readers by foreclosing the idea that the world could ever be radically altered or different. In contrast, speculative fiction estranges the familiar and in doing so, enables readers to step out of modernist, humanist, anthropocentric and realist frames of reference to “question the ontological basis for realities” (Melzer, 2006, p. 6). Through construction of possible future worlds, such fictionalised texts can enable imaginative responses to current crises and provide a valuable resource for critical inquiry and theory-making (Beyes, 2009; De Cock, et al., 2019; Pick, 2017).

The literary genre of feminist speculative fiction is particularly suited to investigating “questions of the posthuman” because these stories “allow us to concretely imagine bodies and selves otherwise” (Vint, 2007, p. 19). Their power does not reside in its ‘truth’ telling abilities or meaning, but rather in the affects, interconnections and disruptions that such stories enable, as part of the thinking process. Posthuman literary theory draws on ecocriticism, posthumanism and speculative fiction (de Freitas & Truman, 2020; Gomel, 2011; Lau, 2018; Vint, 2007). Feminist science studies scholar, Donna Haraway (2013, 2016), has demonstrated how this acts as a method for conceiving alternative visions of subjectivity, such as the cyborg, and provides a conduit for thinking critically and imaginatively about the technologically mediated subjects that we are becoming. Haraway (2013) sees these stories as radical ‘tools’ which instigate “an adventure in worlding…[an] adventure of thinking” (p. 5)—elements which too frequently are missing in business and organisational scholarship (De Cock, et al., 2019). Speculative imagination thus provides a “critical site of engagement” (Vint, 2007, p. 20), which not only dramatizes present crises, such as climate change and pandemics, but also provides a space where “models of possible future selves are put forward as possible sites for identification on the part of readers” (p. 20). This can be used to enable disruption of normative, hegemonic representations of the human subject and to open up alternatives.

Braidotti’s (2019a, 2019b) work is also relevant here because it seeks to displace normative Western conceptions of the autonomous and rational individual and imagine posthuman alternatives through the creative device of figurations. A figuration is a theoretically informed and politically motivated image that “illuminates the complexity of on-going processes of subject formation” (Braidotti, 2019a, p. 85) without recourse to an exclusionary, universal model of subjectivity. Unlike metaphors which are static and representational, figurations are multi-layered dramatizations that enable analysis of power and provide a form of resistance. They are processual works-in-progress, and not just ideas or symbolic images. Figurations can therefore be used to bring forth alternative images of the ethical subject as a dynamic and constantly changing entity that is continually becoming through embodied encounters with others. We begin the next section by explaining the notion of the ethical subject on which posthumanism relies, before using Braidotti’s concept of figurations to explore how human-animal relations in business ethics can be reimagined.

## Posthuman Subjectivity and Affirmative Ethics

Posthumanism begins with a key observation from feminism—the term ‘we humans’ is by no means neutral, but rather denotes a normalised cis-male, individuated, Eurocentric subject that has, historically, overlooked the lives of women. This ethical perspective is founded on feminist critique of humanist, universalist ideals that presuppose ‘Man’ as the measure of all things. Posthumanism builds on an ecofeminist ethics of care (Phillips, 2019, 2020) which calls into question philosophical assumptions that rely on hierarchical and interrelated dualisms to define the human in opposition to nature (Plumwood, 1993). Such dualisms reinforce ‘mastery’ of both nature and women through the interlocking and compounding effects of mind/body, culture/nature, and masculine/feminine binaries. The first terms in these binaries—mind, culture, masculine—have come to signify what is active and human, with the other terms—body, nature, feminine—defined in opposition as passive, and therefore controllable. This reinforces modes of capitalism that negatively and disproportionately impact on women and contribute to the dispossession of peoples from their land and degrades ecologies (Phillips, 2019, p. 1152). By drawing on feminist ethics of care (Noddings, 1984) ecofeminism challenges these tendencies and provides an ethical language through which to motivate action towards more life-affirming and healthy relations between humans, animals and ecologies. Posthumanism extends eco-feminist critique by displacing the human from ‘his’ traditionally elevated position over “the sexualized others (women, LBGTQ +); the racialized others (non-Europeans, indigenous); and the naturalized others (animals, plants, the Earth)” (Braidotti, 2020, p. 466).

Braidotti (2013, 2019a) traces the power relations that characterise interconnected socio-technological transformations, contemporary political, academic and ethical discourses about the nonhuman and inhuman, as well as social and economic practices enabled by globalisation, and their repressive and empowering effects on subject formation. Subjectivity is understood as emerging through multiple and coinciding relational encounters which are characterised by flows of structural power which can entrap and disable—*potestas,* as well as positive forces, desires, values and affects that empower and enable—*potentia* (Braidotti, 2013). This relational ontology has important ramifications for ethical thought because it disrupts the notion that the boundaries of humanity are where ethics starts and finishes.

Braidotti (2013) identifies factory farming as emblematic of the tensions between life and capitalism, laying the ethical deficit directly at the feet of business because animals are: “manipulated, mistreated, tortured and genetically recombined in ways that are productive for our bio-technological agriculture, the cosmetics industry, drugs and pharmaceutical industries, and other sectors of the economy” (2013, p. 8). These technological and scientific advances mean that the very categories of ‘human,’ ‘animal’ and ‘machine’ are shifting and taking on new forms (Braidotti, 2013; Haraway, 2016). Scientific innovations such as genetic modification of plants, stem-cell research, bioengineering and reproductive technologies have turned genetic code into a source of capital.^5^ This repositions humans on “a continuum with non-anthropomorphic, animal or ‘earth’ others” (Braidotti, 2013, p. 95), who are all “caught in the spinning machine of the global economy” (p. 7). Consequently, we are already in the process of becoming posthuman in advanced capitalist societies (Braidotti, 2013, 2019a; Wolfe, 2010). Businesses are having to confront novel ethical challenges that arise from these developments, for example related to cultured meat production and animal cloning (Dilworth & McGregor, 2015). Yet there is profound disquiet about many of these developments as “advanced capitalism both invests and profits from the scientific and economic control and the commodification of all that lives” (Braidotti, 2013, p. 59).

Biogenetic capitalism seeks to extract value from life itself. The concept of *zoe* is crucial to understanding how Braidotti’s politically informed posthuman subjectivity differs from a humanist, anthropocentric concept of equality. Bodies are fully immersed in the ground flow of *zoe* which is “immanent to a network of non-human (animal, vegetable, viral) relations” (Braidotti, 2011, p. 95), and generated from an array of interlocking social, technological, and ecological forces. Instead of equality, Braidotti (2013) refers to *zoe*-centred egalitarianism as a “grounded, situated and very specific and hence accountable perspective” (p. 94). This concept is not against equality but a different way of reacting to and acting within the fields of power that lead to inequality in the first place. Posthuman subjectivity emerges through multiple and coinciding relational encounters within this network of flows of structural power (Braidotti, 2013). This necessitates a “shift towards posthuman ideas of ‘Life’ or ‘*zoe,*’ the non-human” (Braidotti, 2006, p. 37) and recognises the “entanglement of material, bio-cultural and symbolic forces in the making of the subject” (p. 37). Rather than concentrating on moral imperatives for the use and exploitation of animals in organisational contexts, posthumanism displaces established social narratives and power hierarchies that perpetuate forms of domination and pursues a more sustainable ethics that rests on an enlarged sense of interconnection between the self and multiple others (Braidotti, 2013).

## Analysis

Atwood’s trilogy is set in a not-so-distant future that depicts a familiar Western country in transition, following an apocalyptic pandemic and ecological crisis, to new forms of species cohabitation in a post-pandemic world. The distinctiveness of the narrative arises from its rejection of normative conceptions of nature and the presentation of posthuman possibilities, including those that threaten the continuation of human life (Bergthaller, 2010). This includes pre-apocalyptic consequences of a capitalist culture “that sees nature, animals and humans as resources to be exploited by any means in order to sustain the current way of living” (Eriksen & Gjerris, 2017, p. 240), including through factory farming and biotechnological innovation.

Atwood’s *MaddAddam* trilogy offers a discursive device that allows current reality to be problematized and offers insights into more affirmative and sustainable ways of relating to animal and ‘earth’ others. This possibility hinges on the way the novels challenge normative conceptualisations of ‘human’, ‘animal’, and ‘nature’, thereby displacing self/Other human/nature binaries (Ciobanu, 2014). While the trilogy presents an array of humanist, feminist and posthumanist characters, here we focus on a lead female character named Toby. We read Toby as an ethically empowering figuration who is “defined by…[her] relationality and outward-bound interconnections” (Lau, 2018, p. 347). Toby engages in processes of becoming *with* other human and nonhuman animals (including insects), and ecologies to form a trans-species community in a post-pandemic landscape. Toby is placed in situations where she must determine how to act in response to ecological and social changes to make her, and the community’s, future more sustainable and ensure their survival. This involves choices related to consumption, including whether to consume other living beings, some of which are genetically engineered.

We begin the next section with an overview of the novels, followed by analysis of three selected ‘movements’ from Toby’s story. We define movements as dramatized events or moments in the narrative that involve engagement with ethical issues through interactions between human and nonhuman characters. We show how these movements enable imaginative thinking about human-animal relations.

### The MaddAddam Trilogy and Corporate Cannibalism

The first book in Atwood’s trilogy,*Oryx and Crake* (2013), opens in a post-apocalyptic world that has been ravaged by a global pandemic, leaving few human survivors. The immediate cause of this catastrophe is the release of a ‘hot bioform’ (virus) by the disaffected scientist, Crake, whose plan is to wipe out humankind in a ‘Waterless Flood’ and replace them with a new-and-improved bioengineered humanoid species, the Crakers,^6^ who are genetically altered to be vegetarian and cause less ecological harm. *The Year of the Flood* (2009) revisits these same events but from the perspective of an eco-religious cult and resistance group, the God’s Gardeners, whose members attempt to survive Crake’s Waterless Flood. The final book, *MaddAddam* (2013), explores the aftermath of the pandemic, where some remnants of humanity engage in tentative forms of interspecies cooperation in order to survive.

While the Waterless Flood is a catalyst for events in the story, in Atwood’s pre-pandemic world humanity has already arrived at a point of ecological collapse and mass-species extinction. In this dystopian vision of the near future, Atwood employs the trope of corporate cannibalism^7^ in a satirical take on a familiar Western capitalist culture characterised by unsustainable business practices, deregulation and increasing privatisation, a growing rich-poor gap and rampant consumerism. As one character puts it: “We’re using up the Earth. It’s almost gone” (Atwood, 2009, p. 285). It is a world like our own where human, vegetable and animal life, including seeds, plants, animals and bacteria, are “caught in the spinning machine of the global economy” (Braidotti, 2013, p. 7), and genetic code is a source of capital. The novels introduce an array of strange new animal splices that disrupt traditional metaphysical distinctions between humans and other species, most notably, the pigoons. OrganInc Farms is the corporate architect of the pigoon project, the purpose of which is “to grow an assortment of fool-proof human-tissue organs in a transgenic knockout pig host—organs that would transplant smoothly and avoid rejection, but would also be able to fend off attacks by opportunistic microbes and bacteria of which there were strains every year… The pigoon organs could be customized, using cells from individual human donors” (Atwood, 2013, p. 25).

Biogenetic developments are justified as the solution to climate change and harm to animals by elite scientists and businesspeople in Atwood’s narrative. Examples include the growth of monstrous animal-like bodies as a source of human food, like the ChickieNob: “vat grown meat that is a monstrous head-like orifice (without eyes or beak, and allegedly without the ability to feel pain) atop multiple bodies that grow only breast or only drumstick” (Canavan, 2012, p. 142, citing Atwood, 2013, pp. 202–203). Another innovation is the Happicuppa product, made from coffee beans grown on newly deforested swathes of land from “gen-mod, sun-grown, sprayed with poisons” bushes and using machinery which “kills birds” and “ruins peasants” (Atwood, 2009, p.221). The cannibalistic system accelerates, perpetuating “familiar patterns of exclusion, exploitation and oppression” (Braidotti, 2013, p. 96), despite awareness of its destructive ecological impacts. As one character explains, there are:

…more plagues, more famines, more floods, more insect or microbe or small-mammal outbreaks, more droughts, more chickenshit boy-soldier wars in distant countries. Why was everything so much like itself? (Atwood, 2013, p. 298)

Life-threatening connections between the industrial-scientific-military-food complex and the looming apocalypse become increasingly common as the story progresses. As natural resources become scarce, the corporate elite and those who work for them lock themselves away in Compounds, “gated communities…under the protection of the CorpSeCorps, a ruthless and totalitarian private corporate security firm and police force” Bouson, 2011, p. 11). The “non-affluent masses” are restricted to the pleeblands, slum-like areas that are over-populated and dominated by corporate-sponsored criminal activity (p. 11). Bodies are routinely disposed of in the pleeblands by local street gangs that organise kidnappings and assassinations and arrange corpse disposals, harvesting human organs for transplant.

The theme of bodily consumption is reinforced by “the secret of SecretBurgers [which] was that no one knew what sort of animal protein was actually in them” although this is rumoured to involve “running the gutted carcasses” of disposed humans “through the SecretBurgers grinders” (Atwood, 2009, pp. 39–40). Toby is a waitress at a SecretBurgers establishment, where she is subject to the extreme sexual and physical violence of her sadistic boss, Blanco, who sees women as possessions to be used and discarded at will. In her former life, Toby had a degree of class privilege, but after her parents’ death, in debt to the corporations they spent their lives working for, she is forced to ‘disappear’ into the pleeblands. As a result, Toby exists in an increasingly precarious position in a capitalist economy that renders her a disposable body.

Connections are drawn in the narrative between the consumption of meat, violence against animals and sexual violence against women, suggesting they are structurally related (Adams, 2000 [1990]). An example is provided by the Scalies, trapeze artists sheathed entirely in a body suit of shiny green scales who work at the club, Scales and Tails—where men from the “top Corps” go to drink, take drugs and pay to have sex with women who are dressed as fish or other animals. Mordis, the pimp who runs the club refers to Scalies as a “valuable asset” (Atwood, 2009, p.9); Toby’s sadistic boss, Blanco, refers to them as “[a] sex toy you can eat” (Atwood, 2009, p. 500). The disposable bodies of Toby and the Scalies are contrasted with the privileged “virile male adult [who is positioned] as a transcendent subject” (Desmond, 2010, p. 238).

The narrative thus describes a culture not unlike our own where “the use and abuse of animals is… deeply ingrained in the construction of human, particularly male, subjectivity… where man is taken to be superior to woman and animal, adults to children, where some animals are killed and eaten with impunity”, and some humans are accorded a status close to these animals (Desmond, 2010, p. 240). Human and animal bodies who signify difference are rendered disposable commodities in this economy through “being reduced to the use value of one’s body—to a source of labor power or sexual gratification or, in the end, to mere animal flesh” (Ciobanu, 2014, p. 155). This disposability is predicated on ‘sex-species’ hierarchies that privilege male domination and engender a “misplaced sense of superiority” over others (Braidotti, 2013, p. 77). As Braidotti (2013) explains, “[t]he dialectics of otherness is the inner engine of humanist Man’s power, who assigns difference on a hierarchical scale as a tool of governance” (p. 68). Sexualised, racialised and naturalised bodies of those who do not ‘fit’ or can only aspire to the classical image of the knowing (white, male) subject, are relegated to a position of relative inferiority (Braidotti, 2013). Disposal thereby removes diverse others from ethical consideration.

### Movement from Equality Towards In-Disposability

A possible solution to the interconnected issues of disposability and inequality is presented in the form of the God’s Gardeners, an eco-religious group with roots in deep ecology^8^ and animal rights^9^ movements. Difference, in their form of humanism, is a problem to be solved by granting moral and legal equality to those marked as ‘other,’ including animals (Braidotti, 2013). Hence, the Gardeners have strict rules about not eating meat; members take the Vegivows, foregoing the eating of animal flesh. The ethic of sameness which bans the eating of meat of others is evident when Adam One (a leader of the Gardeners) rescues Toby from Blanco and her life as a SecretBurger worker:

Toby was working the morning shift when a strange procession approached along the street… The procession drew up in front of the SecretBurgers booth… “My friends,” said the leader... “My name is Adam One. I too was once a materialistic, atheistic meat eater. Like you, I thought Man was the measure of all things! Yes – I was a scientist. I studied epidemics, I counted diseased and dying animals… But then, one day, when I was standing right where you are standing, devouring – yes! – devouring a SecretBurger, and revelling in the fat thereof, I saw a great light. I heard a great Voice…. It said, Spare your fellow Creatures! Do not eat anything with a face! Do not kill your own soul!... (Atwood, 2009, p. 48).

Adam One’s ethical rationale for not eating meat is based on human-animal kinship, a move which challenges the antagonistic and possessive relationship between ‘self’ and ‘other’ that informs structurally violent and exploitative practices of individuals and corporations that take for granted free access to the bodies of others and ecologies. Humankind’s unchecked destruction of everything good (e.g. nature) is a propelling force for the Gardeners’ actions: “We have betrayed the trust of the Animals, and defiled our sacred task of stewardship” (Atwood, 2009, p. 63). Consequently, their vision of the future is premised on restoring an idyllic Edenic state of harmonious co-existence between species and positions humans as responsible for the protection and care of the natural world.

Much like the myth of human exceptionalism that positions ‘Man’ at the centre of the universe, Adam One’s inversion of advanced capitalism’s promises (growth and progress) is still reliant on the binary division of nature (Animals, insects, ecologies) from culture and society (Humans, technology, knowledge). Any advance in scientific knowledge or technology is a sign of humanity’s ongoing deterioration. As Adam One reminds his followers, “The Fall was ongoing, but its trajectory led ever downward” (Atwood, 2009, p. 224). This ethical philosophy precludes the possibility of imagining alternative modes of engagement with the posthuman present, including the cultivation of affirmative ethical relations with those subjects who are neither entirely natural nor technological. For example, when confronted with the newly developed “hybrid bee,” a genetic splice with “micro-mechanical” insertions, the Gardeners’ label it an “abomination” and an “ethical problem” the resolution of which—and hence the bee’s right to care and protection—is contingent on determining whether or not it is “a true Creature of God or something else entirely?” (Atwood, 2009, p. 329).

While grateful for the Gardeners’ protection, Toby is ambivalent regarding their views, “She didn’t really believe in their creed, but she no longer disbelieved” (p. 116). Her experiences as a disposable body make her sensitive towards power hierarchies and inform her decisions, which are sometimes in opposition to the humanist logic of the male leaders of the God’s Gardeners. This comes to a head after the initial chaos of the pandemic, when Zeb, Toby’s partner and a leader of the Gardeners, decides the priority is to find and rescue Adam One and any others who might be with him. Toby disagrees and instead proposes to save Amanda, a former Gardener woman who has been taken captive by Blanco^10^ and the Painballers and is being raped and tortured to death by them. Zeb argues that “we have to understand that it’s an either/or choice. Amanda’s just one person and Adam One and the Gardeners are many; and if it was Amanda, she’d decide the same thing” (Atwood, 2009, p. 399). Zeb’s statement invokes utilitarian ethics—defining what is morally right as that which produces the greatest good for the greatest number (of humans). While at first appearing reasonable, Zeb’s argument is based on a judgement about the relative worth of Adam One, who represents universalist ideals as a normalised masculine leader, even though he has no idea where Adam One is or whether he is even alive. In contrast, Amanda is positioned as a more disposable subject, even though her whereabouts is known and nearby. Toby’s gendered experiences as a disposable subject make her resistant to Zeb’s logic which renders Amanda less valuable than Adam One in the project of reconstructing humankind after the pandemic. Toby refuses the implicit hierarchical dichotomies underpinning Zeb’s universalist notion of sameness and sets out to rescue Amanda. This act of resistance cuts two ways: “it means both ‘I do not want this’ and ‘I desire otherwise’” (Braidotti, 2019a, p. 166). Toby thereby personifies an expanded, affective subjectivity that is more closely attuned to the posthuman by including what has gone missing—that which is treated as ‘other’ and disposable. For Toby, this means actualising her desire for a future where those marked as disposable do not go missing, a move which rests on an expanded understanding of affect that includes nonhuman life.

### Movement from Individualism Towards Affect

Toby’s interactions with bees illustrate the move from “self-centred individualism” to an “enlarged sense of interconnection between self and others” (Braidotti, 2013, p. 50) that foregrounds an ethics of becoming animated by affect. Haunted by the trauma she has experienced at the hands of Blanco, when Toby is first taken in by the Gardeners she closes herself off and takes no responsibility for others. Noticing her fear, an older Gardener woman named Pilar invites Toby to learn about the bees.

Toby liked Pilar, who seemed kind, and had a serenity she envied; so she said yes. “Good,” said Pilar. “You can always tell the bees your troubles” … Pilar took her to visit the beehives, and introduced her to the bees by name. “They need to know you’re a friend,” she said. “They can smell you. Just move slowly,” she cautioned as the bees coated Toby’s bare arm like golden fur. “They’ll know you next time” … (Atwood, 2009, p. 199)

Pilar’s way of interacting with the bees acknowledges their ability to affect and be affected in turn. Instead of treating the bees purely as a resource for making honey, Pilar recognises their relationship as one of mutual reciprocity and interdependence; an activation of “two ‘becomings-with’” (Haraway, 2016, p. 25). Becoming-with signals the reciprocal co-shaping of a “new metaphysics of subjectivity” (Vint, 2012, p. 44) which does not necessitate the objectification or negation of the other. Without the lover of bees (the beekeeper), the knowledge and expertise of both species, their distribution of tasks and recognition of potential risks, what would remain would be bees, but not as messengers or companion species, as Toby begins to imagine them. This approach denotes a radical form of relationality that is attuned to what others, including the bees, can do. It suggests relational capacities are not confined to the human but are a form of mutual entanglement wherein each interaction is constitutive of the identity of each. Which is to say, every encounter “hybridizes, shifts and alters the ‘nature’ of each one” (Braidotti, 2006, p. 108). This re-configures the human–animal bond as grounded in affectivity and reciprocity, rather than the dominance and separatism that arises from positioning (hu)man over nature.

Affect emerges from relations with others, which for Braidotti (2011) “means openness to others, in the positive sense of affecting and being affected by others” (p. 304). Insects like bees are radically other and thus productively destabilise us, if we let them (Braidotti, 2011). When Pilar dies, Toby communicates the loss, in words, feelings, and in the salt of her tears, which the bees respond to:

Several bees flew around her head, golden in their fur. Three lit on her face, tasting her. “Bees,” she said. “I bring news. You must tell your Queen. Were they listening? Perhaps. They were nibbling gently at the edges of her dried tears. For the salt, a scientist would say.

The bees on her face hesitated: maybe they could feel her trembling. But they could tell grief from fear, because they didn’t sting. After a moment they lifted up and flew away, blending with the circling multitudes above the hives. (Atwood, 2009, pp. 215-216)

As Toby and the bees interact, their bodily boundaries are blurred (they climb into Toby’s nose), through their mutual sense-making (smells and touch), and through their verbal (buzzing and humming) and non-verbal (collective flying) communication. For a moment, where the bees end and Toby begins is an open question. Toby is, ultimately, illiterate in the language of the bees, and her attempts to articulate in human language her inter-relations with the bees is, she realises, almost impossible:

They touch her lips, gather her words, fly away with the message, disappear into the dark. Pass through the membrane that separates this world from the unseen world that lies just underneath it. …Now, Toby, she tells herself. Talking pigs, communicative dead people, and the Underworld in a Styrofoam beer cooler. You’re not on drugs, you’re not even sick. You really have no excuse. (p. 336)

What matters is Toby’s recognition of her own interdependency with the bees as a relation with nonhuman others and the affects this generates, “on subjects and the world” (Braidotti, 2019a, p. 168). Affirmative ethical encounters like this one accentuate *potentia* to resist and transform *potestas*. Toby’s relations with Pilar and the bees enhance her *potentia* and enable her to reimagine her own selfhood. These affirmative affects are not a “‘feel-good’ sort of sentimentality” on the part of the individual, “but rather a rigorous composition of forces and relations” that increases one’s “ability to take in and sustain connectedness to others” (Braidotti, 2011, p. 95). Toby’s becoming an ethical subject thereby involves “cultivating the kind of relations that compose and empower positive passions” (Braidotti, 2011, p. 95), especially those which increase her ability to connect with multiple others, including animals.

Toby’s encounters with other animals, however, are characterised by negativity in the form of an “arrest and blockage that ensue[s] as a result of a blow, a shock, an act of violence, betrayal, a trauma” (Braidotti, 2019a, p. 167). This can be seen in her initial interactions with pigoons, when she shoots one to deter them from accessing and destroying her garden. Toby’s intention is to protect her only reliable source of food, which is grown in the garden, in the aftermath of the pandemic but she is also tempted to eat the pigoon. However, affirmative ethics suggests “the subject’s ethical core should not be defined in terms of intentionality, but as its forces and affects” (Braidotti, 2019a, p. 136). Given that all subjects – human, animal and hybrid – are part of and entangled with the material world, any harm Toby does to others is immediately reflected in the harm she does to herself (Braidotti, 2018). Toby’s act of violence engenders negative effects not only for the pigoons, whose survival is put at risk as their numbers decrease, but also for Toby and her community, whose capacity to relate to this new hybrid species is diminished as a consequence.

### Movement to Imagining New Forms of Becoming

Confronted with the task of survival in a post-pandemic landscape, Toby and the other human and nonhuman members of the community gradually forge links across species. The God’s Gardener doctrine that advocates the restoration of human beings to their purportedly natural role as caretakers and protectors of animals and ecologies is inadequate to this task. Not only does it reinforce the binary distinction between humans and animals, but it fails to acknowledge that “[l]ife lives on regardless of human pretensions and expectations” (Braidotti, 2019a, p. 182). This includes new forms of life that are neither entirely natural nor technological, such as the pigoons and the Crakers. Toby becomes aware of these complexities as she attempts to chronicle the community’s daily struggles in her journal: “She could go further, and record the ways and sayings of the God’s Gardeners for the future; for generations yet unborn…If there is anyone in the future, that is…[but] Maybe acting as if she believes in such a future will help to create it” (Atwood, 2013, p. 166).

Following Amanda’s rescue by Toby and Ren, the two remaining Painballers manage to escape, mistakenly freed by the Crakers, who are both non-violent and sensitive to the suffering of others, and so cannot understand why the humans want to restrain them. The community is also threatened by the pigoons who raid their garden in retaliation for the humans shooting and eating them. With their spliced human brain tissue, the pigoons possess a degree of intelligence and cunning that unsettles the humans. “‘Ever since we turned a couple of them into bacon,’ said Manatee. ‘Frankenbacon, considering they’re splices. I still feel kind of weird about eating them’” (Atwood, 2013, p. 28). The possibility of more generative forms of posthuman relationality, however, cannot be achieved by simply humanising the pigoons (or the Crakers) and granting them ‘rights’ on human terms. Rather this posthuman relationality is contingent on an expanded notion of interdependence with multiple others and the living ecological systems of which they are part (Braidotti, 2019a).

Because the human-pigoon relationship is characterised by harmful events and experiences (*potestas*) there can be no return to Edenic innocence. For Toby, overcoming the pain of past encounters begins with reimagining her relationship to the pigoons, thinking of them not as objects or food (‘spare-ribs’ and ‘bacon’), but as subjects who possess agency. This openness to animal-others foregrounds a middle-space between humans and nonhumans and creates possibilities for new relations and values to emerge through embodied interaction (Braidotti, 2013). This takes the form of an alliance between the pigoons, humans and Crakers, who begin to work together to overcome the destructive threat of the remaining Painballers.

Toby’s community, which now consists of several species, is cautiously burgeoning. There is an inter-species pregnancy (human-Craker), alternative forms of communication (telepathy) between Crakers and pigoons, humans agree not to eat pigoons, and there is a possibility Crakers will learn to read. What might emerge as part of this new milieu of diverse posthuman subjects and changing bodies is neither self-evident nor pre-given. Instead it is an ongoing experiment shaped by immanent interconnections and the creation of narratives that recognise difference. “‘I am writing the story,’ she [Toby] says. ‘The story of you, and me, and the Pigoons, and everyone.’” (Atwood, 2013, p. 456). By the end of the trilogy, Toby’s writing has begun to intermingle with that of Blackbeard, her Cracker protégé whom she has taught to read and write. Toby initially worries that the effect of these technologies on the Crakers could result in “Rules, dogmas, laws… How soon before there are ancient texts they feel they have to obey but have forgotten to interpret?” (Atwood, 2013, p. 250). Yet language also functions as a tool of the creative imagination, a “thing of hope” (Atwood, 2013, p. 474) for the community. There is no Utopian ending; the group’s survival is tentative at best. As Blackbeard explains, “*Hope* is when you want something very much but you do not know if that thing you want will really happen” (p. 355).

The ‘Book of Toby,’ so named by the Crakers, enfolds within it the possibility of a sustainable future that is characterised by multispecies flourishing. Ciobanu (2014) identifies exactly this ethical component in the novels, stating: “[t]his, then, is the posthuman that Atwood offers. The posthuman is a new way of inhabiting our humanity rather than a new-and-improved version of the human” (p. 160). By weaving together different voices and stories—Craker, human, pigoon, bees, and soon-to-be human-Craker hybrids—Toby’s story denotes an ethical subject that is ceasing to be the conflict-driven, normatively individuated, detached human being and is instead in an immanent and continual process of becoming *with* animal, insect, machine and earth others in what is ‘our’ shared territory (Braidotti, 2013). This ethical view emphasizes the role of the imagination in envisaging and enacting new cross-species relations.

The three movements presented above illustrate how posthuman affirmative ethics is enacted in posthuman contexts. They show how figurations can be used to imagine affirmative responses to the precarious posthuman present by breaking with traditional notions of the separate and sovereign human subject. In the discussion that follows, we further clarify the contribution of affirmative ethics by explaining how it enables revision of notions of equality in business ethics.

## Towards a Posthuman Affirmative Business Ethics

We began this paper by posing the question: what would posthuman affirmative business ethics look like, and what changes in thinking and practice would this involve? An affirmative ethical perspective acknowledges the imbricated relations between humans and diverse others as a basis for (re)immersion in the flow of life (*zoe*). We begin this discussion by returning to the issues introduced at the start of the paper—related to contemporary crises in human-animal relations and the need for business ethics to address them. We then elaborate further on our three movements: equality towards in-disposability; individualism towards affect; and imagining new forms of becoming. Through this, we clarify how an expanded concept of subjectivity enabled by posthuman affirmative ethics enables revision of the notion of equality.

Braidotti (2020) observes that the solution to the COVID-19 crisis as a (hu)man-made disaster caused by interference with the lives of multiple species and their ecologies, has focused on vaccines. But this biomedical ‘solution’ is actually a symptom of the problem. She suggests the pandemic provided the conditions for us to clearly see the posthuman condition by making visible connections between ecological crises (the Sixth Extinction) and the excesses of advanced capitalism (the Fourth Industrial Age). Braidotti further states: “viruses born of human interference with animals and environmental sources, such as COVID-19, are anthropogenic and hence discriminate as much as humans do. They act as indicators of massive social inequalities, which dominant neo-liberal political classes are intent on denying” (2020, p. 466). These recent comments by Braidotti reinforce the need for posthuman thinking in business ethics.

The relevance of Braidotti’s arguments can be illustrated by returning to the ‘problem space’ of factory farming. Posthumanism enables connections to be made between ethical concerns about suffering caused by industrial animal farming and associated ecological destruction, with the ethics of treating some human lives as disposable. This analytical process begins by exploring the *potestas* that create inequities which affect multiple others. Recent organizational research that engages with human-animal relations in this way includes Hamilton and McCabe’s (2016) study of how workers’ subjectivities are managed to minimise affects that arise from their encounters with animals in meat-processing plants. A further illustration is provided by Clarke and Knights (2021) whose research into dairy farming and veterinary practice seeks to challenge the “anthropocentric segregation of humans and animals” (p. 1) by showing how humans obscure their recognition of the suffering of animals despite professional ethical codes which advocate against this, in the interests of business. However, the potential of posthumanism for business ethics also relies upon extending understanding of *potentia*—the positive forces, desires, values and affects that empower and enable. To do this, we return to the three movements introduced in our analysis.

First, regarding the movement from equality to in-disposability. Posthuman affirmative ethics rejects the “the unitary, humanistic vision of a fixed and self-transparent subject” (Braidotti, 2006, p. 208). Instead, it depends on an open, dynamic notion of subjectivity as becoming. Affirmative ethics invites us to imagine a future which is open to diverse others, and to acknowledge our shared vulnerability in relation to them. This includes a vulnerability that is shared with animals and other living matter that are routinely treated as disposable. Yet such inequities are not experienced equally, for while “we *are* in this together” (Braidotti, 2011, p. 218), subjects “are-not-one-and-the-same” (Braidotti, 2019a, p. 54). Posthuman affirmative ethics thus remains politically sensitive to differential effects of power on people and other species.

The notion of movement towards in-disposability provides a different basis for understanding equality in organisations. The notion of equality in business ethics relies on managerial discourses that construct a false dichotomy and embody a male standard against which notions of ‘sameness’ and ‘difference’ are judged—these “terms are inter-dependent, in that one can only be different in so far as one is not the same as the other” (Liff & Wajcman, 1996, p. 80). This logic places emphasis on achieving ‘equal’ treatment for others by extending to them a rights-based ethics that seeks to ensure they are treated in the same way as the unitary, masculine subject. However, it fails to question or destabilise “the schema of the human” (Wolfe, 2010, p. 99), especially the presupposition that nature is passive, waiting to be granted ‘rights’ or ‘justice’ by humans who possess consciousness. As Braidotti (2013) argues, “the principle of moral and legal equality” is thereby extended to animals, rather than respecting their otherness (p. 79). This reinforces a dualistic division between humans and animals “by benevolently extending the hegemonic category, the human, towards others” (Braidotti, 2013, p. 79). Posthuman affirmative ethics advocates an alternative basis for equality through *zoe*-centred egalitarianism. Such a perspective enables diversity agendas, which have sought to encompass a wider array of stakeholders and considerations into business ethics, to be extended by introducing a more “radical [vision] of the subject” (Cornelius et al., 2010, p. 6).

Second, the movement from individual intentions and outcomes towards affect and potentialities (*potentia*) is enabled by the development of co-constituted mutual human and nonhuman subjectivities. Affect implies that bodies and emotions are open to each other. When we are open and vulnerable our boundaries are understood as permeable. Through this, we become more open to the suffering of multiple others. Vulnerability thereby engenders a heightened sense of responsiveness and response-ability to and with—rather than for—the other. It animates the *potentia* of affirmative ethical encounters. This movement means the ethical concept of equality becomes grounded in vulnerability (see also Phillips, 2014, 2019), which is extended to encompass multiple others. It makes vulnerability and the precarity which arises from it, a point of connection and thus affirmation. Ethics is thereby “defined as the pursuit of affirmative values and relations” (Braidotti, 2019a, p. 136). The posthuman subjectivities that emerge from this movement enable appreciation of how practices such as factory farming damage humans by lessening our capacity to encounter and sustain positive (*potentia*) affects.

Third, regarding the movement to imagining new forms of becoming, Braidotti (2013) puts the argument eloquently: “[t]he future is an active object of desire that propels us forth and motivates us to be active in the here and now of a continuous present” but also, this active desirable future requires both “resistance and the counter-actualisation of alternatives” (p. 192). As this statement suggests, posthuman affirmative business ethics relies upon collective activism, rather than individualistic rationalism. The imagining of alternatives is what makes posthumanism affirmative; possible futures become through the capacity to affect and be affected through encounters with multiple others. This approach to subjectivity relies upon relations based on empathy and connection and resists foreclosure by remaining open-ended.

As the COVID-19 pandemic has highlighted, harms done to animals in biopolitical spaces including factory farming have reciprocally detrimental effects on humans as well as other animals. This generates mutually destructive and, we suggest following Braidotti, ultimately unsustainable relations with multiple others by diminishing the capacity of humans to act affirmatively. Our contribution has been to show how posthumanism provides an alternative philosophical perspective through which human-animal relations can be reimagined in business ethics. This requires a move away from human/anthropocentric ethical perspectives through *zoe*-centred egalitarianism, enabled by introducing alternative figurations of the subject.

## Conclusion

Posthuman affirmative ethics rejects the notion that other human and nonhuman bodies are disposable and instead seeks to develop an expanded, affective subjectivity that is founded upon affective, material and immaterial connections to multiple others. This “collaborative and interconnected ‘we-are-in-this-together’ kind of subject” (Braidotti, 2017, p. 23) engenders opportunities to develop affirmative ethical values centred on affectivity and relationality. Thus, while posthuman affirmative ethics does not displace humanism, it refuses to accept its limitations and seeks to establish a different relationship to, and with, nonhuman life. Yet posthuman subjectivity is not “restricted to bound individuals” and instead is a “cooperative trans-species effort” (Braidotti, 2019b, p. 33). It recognizes that resistance to oppressive and exploitative practices, through a desire to remake the world, starts with a different model of ethical subjectivity.

Feminist speculative fiction provides imaginative resources that can be used to develop notions of subjectivity where *zoe*-centred egalitarianism can take root. By “elaborating [on] posthuman ethical scenarios, the genre confronts its readers with the fact that the Universal Man is already dead” (Gomel, 2011, p. 352). The critical imagination that feminist speculative fiction enables provides a crucial means of “retraining readers to think outside anthropocentric and humanistic habits” (Braidotti, 2019a, p. 133). Such reimagination relies upon the construction of mobile figurations that can be used to build better futures for all that lives.

Shared affective states, such as vulnerability, are amplified by crises such as pandemics, climate change, ecological degradation, and the threat of extinction. A final example of a figuration is provided by Braidotti (2020) who concludes her article with an image of a genetically modified bat-boy by Melbourne artist Patricia Piccinini. The bat-boy has the body and some facial features (e.g. large ears) of a bat and the face of a human child. The bat-boy hangs upside down with his wings swaddling his fragile body, gazing upwards with partially obscured human eyes. Next to the image is a slogan: “Is there room in our hearts?” This image asks us to contemplate how far our ability to imagine ourselves as posthuman subjects can be extended in order to respond affirmatively to the future.

## Footnotes

1 Research confirms that human effects on the environment, associated with climate change and industrial food practices, enable the conditions for pandemics to flourish and that encouraging biodiversity will reduce the likelihood of infectious diseases (Keesing et al., 2010).

2 Books and documentaries such as Saffron-Foer's (2009) *Eating animals*, Shah’s (2017) *Pandemic* and documentaries such as *Game Changers* (2018), and *Food, Inc*. (2008) are critical of global capitalist animal food production practices.

3 Scientific, business and health evidence are mounting about COVID-19’s impact on diverse disadvantaged groups. For examples of the negative impact on women see: https://www.mckinsey.com/featured-insights/future-of-work/covid-19-and-gender-equality-countering-the-regressive-effects; and negative health impacts on ethnic groups: https://www.bmj.com/content/369/bmj.m2122.

4 ‘Nonhuman animals’ is the preferred term in human-animal studies for animals that are not human. The use of this term is a political reminder to question the false divide between humans and animals. Here, we use the terms ‘animals’ and ‘nonhuman animals’ interchangeably.

5 For example see: Grens (2018) and Wolf et al. (2019); and ‘China’s CRISPR push in animals promises better meat, novel therapies, and pig organs for people’, *Science.* https://www.sciencemag.org/news/2019/07/china-s-crispr-push-animals-promises-better-meat-novel-therapies-and-pig-organs-people [Accessed November 4, 2019].

6 The Crakers are ‘monstrous’ hybrids; spliced with hares and rabbits, baboons, octopuses, and cats. They are designed with an innate distaste for meat but can eat the plants that will survive in the post-apocalyptic world that Crake plans for them. Atwood represents them as a peaceful, emasculated and slightly naïve species, who are engineered to flourish in a globally warmed, fatally polluted world.

7 See Bouson (2011) for further discussion of how Atwood engages the trope of corporate cannibalism as a discursive device in *Oryx and Crake* and *Year of the Flood* to call attention to the potentially disastrous implications of unmitigated corporate control and consumerism.

8 Deep ecology is founded on a view of “the evolutionary kinship of all species and the ethical obligations it entails” (Bergthaller, 2010, p. 739).

9 Rights-based discourses argue for the ‘becoming human’ of animals to emancipate animals from their secondary, and hence pejorative, position within society.

10 Blanco, Toby’s main nemesis in *Year of the Flood*, remerges in *MaddAddam* after becoming even more violent due to his experiences in a penal facility where, before the Flood, criminals were organised to kill each other off in ‘Painball!’, a live television show.
